# Platlet Rich Plasma (PRP) Improves Fat Grafting Outcomes

**Published:** 2013-01

**Authors:** Ali Modarressi

**Affiliations:** University Hospitals of Geneva, Geneva, Switzerland

**Keywords:** Platlet rich plasma, PRP, Fat, Graft, Outcome

## Abstract

Autologous fat transfer offers many qualities of a ideal soft tissue filler. Main advantages of fat grafting ensue from the fact that the lipoaspirate tissue is an abundant source of regenerative pluripotential cells. However, the reported rates of fat cell survival vary greatly in the medical literature (10-90%). Different techniques of harvesting, processing, and reinjecting the fat cells are so claimed to be responsible for these differences, without any agreement concerning the best way to process. To address this important disadvantage, we propose the addition of autologous platelet rich plasma (PRP) which is known as a natural reservoir of growth factors stimulating tissue repair and regeneration. This approach is completely autologous and immediately employed without any type of preconditioning. Platelets rich plasma (PRP) preparation included bleeding of 8 ml of blood from patient’s peripheral vein in Regen Lab© tubes containing sodium citrate anticoagulant. The whole blood was centrifugated at 1500 g during 3 min. As Regen-tubes contained a special gel separator, 99 % of red blood cells were discarded from the plasma at the bottom of the gel, and >90% of platelets were harvested in 4 ml of plasma on the top of the gel, called the platelet-rich plasma (PRP). The purified fat prepared by Coleman technique was mixed with different amount of PRP for in vitro, in vivo (mice) and clinical experiments: >50% of PRP for skin rejuvenation, superficial scars correction, infraorbital region, ..., and for 20% of PRP with 80% of purified fat for deep filler indication (nasolabial folds, lips, or soft tissue defect). In vitro studies demonstrated that PRP increased fat cells survival rate and stem cells differentiation. Animal models showed that fat graft survival rate was significantly increased by addition of PRP. Several clinical cases confirmed the improvement of wound healing and fat grafting survival in facial reconstruction and aesthetic cases by association of fat grafting with PRP. The addition of PRP to fat grafts represented many advantages with a simple, cost-effective and safe method. In addition to its booster effect on fat grafts, PRP had a rejuvenation capacity per se. It is also used on nappage technique, on mask and as a temporary regenerative filler in combination with thrombin. So we consider the addition of 20% PRP to fat grafts offers a better fat grafting survival, a less bruising and inflammation reaction, and easier application of fat grafts due to liquefaction effect of PRP.

## INTRODUCTION

The importance of addressing the volume loss is becoming increasingly evident to cosmetic surgeons and patients. This volume loss can be corrected through several means, including tissue repositioning (e.g. facial lifting), implants (e.g. malar implants), synthetic fillers (e.g. hyaluronic acid) or autologous tissue.

More recently, autologous fat grafting has come to be considered an ideal filler, becoming a clinical reality in aesthetic medicine and surgery. The success of fat grafting is thought to originate the abundant source of regenerative multi-potent cells in particular, Adipocytic derived Stem Cells (ADCs). These cells are all capable, of integrating into host tissue and to secrete an important orchestrated quantity of cytokines and growth factors including vascular growth factor (VEGF), hepatocyte growth factor (HGF), insulin-like growth factor (IGF), platelet derived growth factor (PDGF), and transforming growth factor-beta (TGFβ).^1^

Main advantages of fat grafting include (i) A long lasting result contrary to the synthetic resorbable products, (ii) Prevention of granuloma and allergic reactions which could be provoked by the permanent products injection, (iii) A natural consistency and (iv) An improvement of cutaneous and subcutaneous trophicity.

On the other hand, disadvantages of autologous fat grafting include (i) Its complexity of use, requiring a more important learning curve with regard to the prepared products, (ii) The morbidity and the necessity of donor site, that sometimes could not be enough, and mainly (iii) The unpredictability of the remaining volume by partial uncontrolled absorption of the fat transplant.^2^ To address this latter important disadvantage, we propose the addition of autologous, platelet rich plasma (PRP), which is known as a natural reservoir of growth factors. 

**AUTOLOGOUS FAT GRAFTING**

The transplant of autologous fat tissue, so called lipomodelage, liposculpture or lipofilling has been known since the early of 20th century. The principle of the technique is to transfer patient’s own fat tissue from a donor site (e.g. abdomen, flanks, thighs) to a site where there is a volume deficit. Its first indications were for aesthetic surgery of the face,^3^ and more recently in hands.^4^ Fat grafting is also useful for tissue loss due to an accident, operation, congenital disease or lipodystrophy.^5^^, ^^6^ In addition to a volumizing effect, the injected fat leads to a neoangiogenesis effect^7^ improving the cutaneous elasticity, and to an antiaging effect.^1^ This technique is thus also recommended for wound healing,^8^ scar reduction,^9^^,^^10^ radiodermatitis treatment^11^ and correction of acne scars.^12^ During the last decade, fat grafting has been used more and more frequently for breast reconstruction and augmentation.^13^^,^^14^

Despite Neuber’s use of fat transfer in 1893, the first description of the fat transplantation by infiltration dates to 1962 by Miller. Given the little satisfactory long-term results, this technique did not have the expected success and other numerous methods were described. At present, the most used method for the fat harvesting, purification and infiltration is the one described in detail by Coleman in 1986,^15^^,^^16^ This method takes into account the fragility of fat cells during the various steps of the treatment.

The survival rate and longstanding results depend partially on indications and patients but mostly on surgical technique. However, even with the best surgical technique, the fat grafting survival is unpredictable, with a variable resorption rate reported throughout the literature (10% to 90%). Different techniques have been proposed to improve the survival rate of fat grafting and its predictability. The most efficient technique proposed until today is to highly enrich the grafted tissue on mesenchymal stem cells. However most of these technique are time consuming, expensive with significant harvested fat loss, and the results still remain unpredictable.

Another approach to improve the fat grafting results could be stimulation of transplanted tissue by growth factors. Unfortunately, the exogenous and synthetic growth factors treatment have not provided the desired expected results in clinic (e.g., wound healing treatment, bioengineering). One of the reasons is the protein fragility and instability of growth factors. Recently, autologous platelets considered as a natural reservoir of growth factors has been used for different pathologies. So, we suggest that its addition to fat grafts could be a solution to boost stem cell survival, multiplication and differentiation to improve the long standing results of lipofilling efficiently and simply.

**PLATELETS RICH PLASMA (PRP)**

Platelets are enucleated circulating blood particles that derive from the fragmentation of megakaryocytes. They circulate in an inactivate state until they come into contact with endothelia damage areas. Platelets work via the degranulation of the α-granules in platelets, which contain the synthesized and pre-packed growth factors. The most potent ones in restoring damaged tissues are PDGF, TGFβ, IGF, VEFG and endothelial growth factor (EGF).

The synthesized growth factors directly bind to the surface of cell membranes to stimulate hemostasis and normal healing. They induce internal cellular signaling that activates angiogenesis, cell proliferation, cell differentiation and new matrix formation for tissue repair. Platelets are therefore a natural reservoir of growth factors that could be used to regenerate tissues. Previous topical growth factor studies have shown that synthetic human platelet-derived growth factors could be an efficacious treatment for wound healing. However, as those synthetic proteins presented some limits for clinical use, a newer treatment, autologous platelet-rich plasma (PRP), has been developed. It represents a greater similarity to the natural healing process as a composite of multiple growth factors. It is safe due to its autologous nature, and is produced simply as needed from the patient’s blood.

After 30 years of PRP clinical application to stimulate bone regeneration and wound healing, autologous PRP is actually recognized as a new tissue engineering element and a developing area for clinicians and researchers that helps healing of soft and supportive connective tissues.

The benefit and safety of PRP is documented in more than 5,000 studies where the authors observed enhancement of bone regeneration,^17^^-^^19^ wound healing,^20^^-^^22^ tendon and cartilage healing,^23^^-^^25^ corneal healing ^26^ and skin rejuvenation.^27^ PRP is so used more and more often in the plastic, reconstructive and aesthetic surgery fields.^28^^-^^30^

**FAT GRAFT AND PRP**

Recently, there has been increased interest in the co-application of PRP and fat grafts. The live fat tissue is revascularized at the transplantation site within 48 hours. During this time, it is fed by diffused material in the plasma. In contrast, non-viable tissue is removed by macrophages, leaving behind fibrotic and cystic changes. The main obstacles preventing permanent augmentation are partial absorption and ischemia of the transplanted fat tissue, which often necessitate multiple transplantations. Then, the quality of transplanted tissue becomes highly dependent on the healing process, restoration of vascularization and adipocyte differentiation. The reported rates of fat cell survival vary greatly in the medical literature, and different techniques of harvesting, processing, and reinjection of fat cells are claimed to be responsible for these differences.

However, there is no consensus concerning the best way to process the harvested fat before reinjection. Based on recent literature, we hypothesized that adding PRP to fat preparation may be a reliable way to bring appropriate nutrient at the early moments of transplantation to improve fat survival and render the result more predictable. PRP releases the native growth factors in their biologically determined ratios at the treatment site. Released growth factors stimulate angiogenesis, cell differentiation and proliferation leading to the reconstitution of the tridimensional matrix that allows the rearrangement of adipocytes into the correct 3D organization. This approach is completely autologous and immediately employed without any type of in vitro preconditioning or media complement.

In a series of in vitro studies, it has been demonstrated that PRP increases fat cells survival rate and stem cells differentiation.^28^^,^^31^ Nakamura *et al.* showed that fat graft survival rates are significantly increased in rats.^32^ Finally, several clinical cases have been reported to improve wound healing by association of fat grafting with PRP.^33^ There is also some successful cases of facial reconstruction with fat grafting and PRP.^34^ This association has recently been also described for aesthetic cases.^35^

**FAT GRAFTING WITH PRP TECHNIQUE**

Firstly, patients must be assessed correctly during initial consultation. Volume loss occurs in various patterns according to its cause; diffuse volume loss after massive weight loss is different from localized volume loss only in nasolabial, infraorbital region or lips due to aging. Skin texture and thickness is another point to be evaluated before treatment. Patient information is very important because the agreement between patient’s expectation and the result that can be offered by the treatment is a guarantee of satisfaction.

Adipocytes have short lifespans once removed from the body, and they do not react well to excessive handling, refrigeration or major trauma during tissue collection or processing. The fat graft resorption is the main drawback^36^^,^^37^ which could be dramatically reduced by using good technique.

The method described by Coleman advances the principle that the fat transplants have to survive and be revascularized. Coleman *et al.* recommends a small quantity of fat injection in fine layers to increase the proportion of fat graft surface area to receptor bed.^15^ The total procedure could be realized with local or general anesthesia, according to patient/physician preference and the importance of fat volume previewed to be grafted.

**DONOR SITES**

The most common donor site in clinical practice is the abdomen, but the fat could be harvested from any location that presents adequate non-fibrous fat flank, thigh, and medial knee which is patient-specific, and dependent on patient/physician preference. There is no compelling evidence regarding harvest site and efficacy of fat grafting.^38^

**FAT HARVESTING**

Adipocyte viability decreases with increasing negative suction pressure.^39^ Thus, mechanical liposuction by machine should be avoided (~500 mmHg), and only manual harvesting offers a satisfied fat graft quality.^40^ Low pressure vacuum, created by a 2 ml withdrawing plunger of a 10 ml Luer Lock® syringe, gives the best result. The fat harvesting is performed with a blunt cannula connected to 10 ml Luer Lock® syringe. The ideal cannula combines efficient collection of fat parcels with minimal neurovascular damage. The most used is a blunt tip cannula with a single distal opening of 3 mm diameter. For small and precise fat grafting (e.g. suborbital region), we suggest the use of 1.65 mm cannula.

**FAT PURIFICATION**

**Fig. 1:**
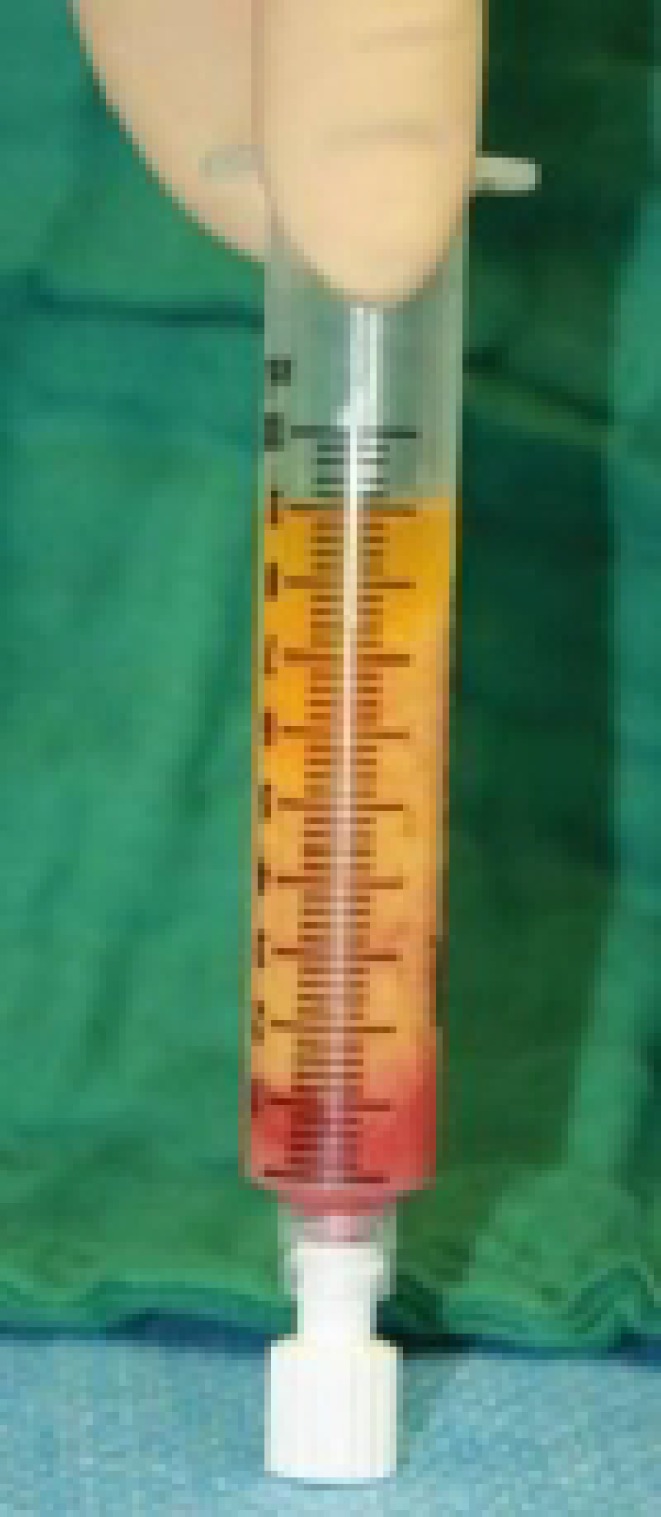
Harvested fat after centrifugation: 1) Upper part: Oil from damages adipocytes, 2) Middle part: Purified fat, and 3) Lowest part: Red cells, cell’s debris and liquids

The ideal method for fat purification would separate blood, infiltration fluid, and cell debris from healthy adipocytes with minimal trauma (Figure 1). This particular step is the most debated part of the fat grafting procedure, subjected to intense scrutiny without, however, a definitive solution. While various methods for separating out fat have been described, none has been determined to be superior to the others, but it is accepted that techniques involving less manipulation may have better outcomes including (i) Sedimentation: Aspirate material stands for 30 minutes to 1 hour, which separates it into its various components;^41^ (ii) Washing: aspirate fat is washed with 5% glucose solution, 0.9% normal saline, or sterile water,^42^ (iii) Filtration: Harvested fat is placed on sterile gauze over sterile cup, washed with ringer’s lactate and dried before loading into syringes;^43^ (iv) Centrifugation: Harvested fat is centrifuged 3 minutes at 3000 rpm. This method separates fat from substances that increase degradation, and concentrates adipocytes and stem cells per milliliter of fat transplanted.^44^ It is the most rapid and clean method.

Various studies assessed the impact of centrifugation on fat transfer, and most of have concluded that centrifugation, unless conducted at very high speeds, does not adversely affect adipocyte viability.^45^^,^^46^ Coleman *et al.* suggests 3000 rpm for 3 minutes, but 1 minute of centrifugation is as efficient with less harm to fat cells.^15^

**PRP PREPARATION**

Today, there are different techniques for PRP preparation in the market. Since 2003, RegenLab has developed a new technique to prepare autologous PRP from whole patient blood. This is a simple and safe method to realize in the operating room while maintaining low cost. It requires no specialized skill and a small amount (8 ml) of patient’s blood is enough. In comparison to other methods, this technique has also been shown to offer the best platelet concentration and survival, with highest growth factor secretion.^47^^,^^48^

This method of PRP preparation has already shown good results for bone regeneration^17^ and skin rejuvenation^49^. As it is a safe, efficient, simple and cheap system for a better and predictable fat grafting, in our clinical practice, we use this RegenLab PRP preparation method.

**Fig. 2:**
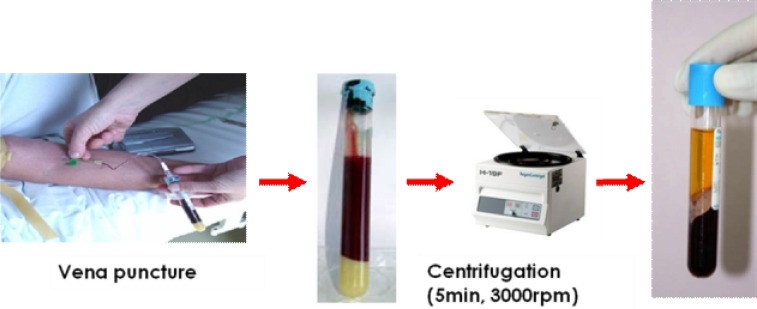
PRP preparation with RegenLab kit: 1) 8 ml of blood vena puncture in Regen-tube: Upper part=total blood, lowest part=gel separator; 2) Regen-tube after 5 minutes of centrifugation on 3000 rpm: Upper part=PRP, middle part=gel separator, lowest part=red cells and debris

Eight ml of blood is withdrawn from patient’s peripheral vein in Regen-tubes containing sodium citrate anticoagulant. The whole blood is centrifuged at 3000 rpm during 5 min. As Regen-tubes contain a special gel separator, 99% of red blood cells are discarded from the plasma at the bottom of the gel. Platelets and white blood cells are pellet on top of the gel and re-suspended in plasma by gently mixing the tube (Figure 2). The 4 ml of cell suspension is called the PRP.

**FAT AND PRP MIXTURE**

The purified fat by centrifugation is mixed through a 3-ways connector with 20% of PRP. According to our in vitro experiments, the 80% fat/20% PRP seems to be the optimal rate for cell proliferation and survival. 

**FAT/PRP INJECTION**

**Fig. 3:**
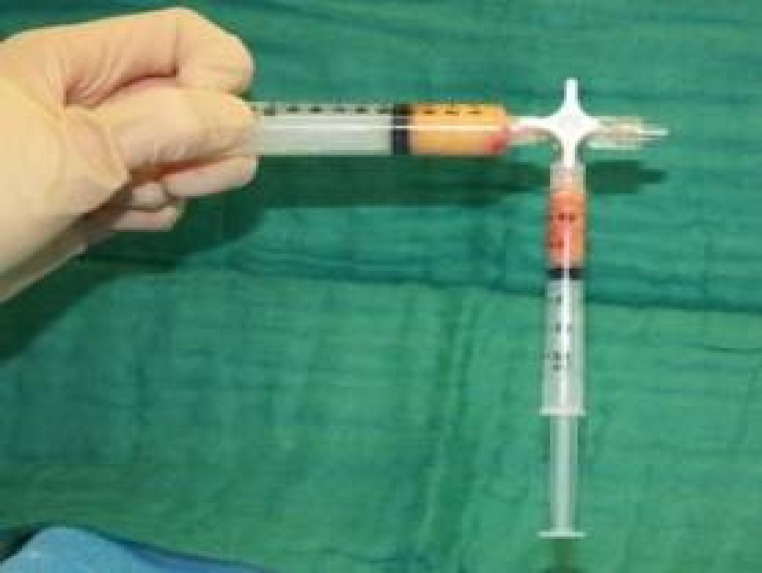
Purified fat transfer from 10 ml Luer Lock® syringe to a 1 ml Luer Lock® syringe through a 3-ways connector.

The fat/PRP mixture is transferred from 10 ml Luer Lock® syringes to 1 ml or 3 ml Luer Lock® syringes via a 3-ways connector (Figure 3). It is important to use smaller syringe, because the fat placement is more precise. For fat placement, special blunt cannula (0.75 mm to 1.65 mm) is connected to the 1 or 3 ml syringes.

As suggested by Coleman *et al.*,^15^ fat is injected in small parcels and thin strips in several layers. Before injection, it is recommended to create some tunnels, especially in nasolabial region or in scars, to release fibrotic tissues. The fat graft is then placed by a withdrawing way.

## CONCLUSION

A basic principle of aesthetic surgery is to replace “like with like”. Autologous fat transfer offers many of qualities of an ideal soft tissue filler: It is biocompatible, inexpensive, readily available, non-migratory with long term results. However, even with the best technique, the survival rate is still quite variable and unpredictable. The addition of PRP to fat grafts represents several advantages with a simple, cost-effective and safe method. We recommend this combination for all fat grafting, but especially for aesthetic purposes. In addition to its booster effect on fat grafts, PRP has a rejuvenation capacity per se. It is also used on nappage technique for skin or hair regeneration. To summarize, we conclude that the addition of PRP to fat grafts offers several advantages including (i) Better fat grafting survival, (ii) Less bruising and inflammation, and (iii) Easier application of fat grafts due to liquefaction effect of PRP.

## CONFLICT OF INTEREST

The authors declare no conflict of interest.

## Footenote

Please cite this paper as:

Modarressi A. Platlet Rich Plasma (PRP) Improves Fat Grafting Outcomes. World J Plast Surg 2013;2(1): 6-13.
